# Deep neural network-based classification of cardiotocograms outperformed conventional algorithms

**DOI:** 10.1038/s41598-021-92805-9

**Published:** 2021-06-28

**Authors:** Jun Ogasawara, Satoru Ikenoue, Hiroko Yamamoto, Motoshige Sato, Yoshifumi Kasuga, Yasue Mitsukura, Yuji Ikegaya, Masato Yasui, Mamoru Tanaka, Daigo Ochiai

**Affiliations:** 1 Department of Pharmacology, School of Medicine, Keio University, Tokyo, 160-8582 Japan; 2 Department of Obstetrics and Gynecology, School of Medicine, Keio University, 35 Shinanomachi, Shinjuku-ku, Tokyo, 160-8582 Japan; 3 Department of Systems Design Engineering, Faculty of Science and Technology, Keio University, Kanagawa, 223-8522 Japan; 4 Graduate School of Pharmaceutical Sciences, The University of Tokyo, Tokyo, 113-0033 Japan; 5 Institute for AI and Beyond, The University of Tokyo, Tokyo, 113-0033 Japan; 6 Center for Information and Neural Networks, National Institute of Information and Communications Technology, Suita, Osaka 565-0871 Japan

**Keywords:** Computational biology and bioinformatics, Medical research

## Abstract

Cardiotocography records fetal heart rates and their temporal relationship to uterine contractions. To identify high risk fetuses, obstetricians inspect cardiotocograms (CTGs) by eye. Therefore, CTG traces are often interpreted differently among obstetricians, resulting in inappropriate interventions. However, few studies have focused on quantitative and nonbiased algorithms for CTG evaluation. In this study, we propose a newly constructed deep neural network model (CTG-net) to detect compromised fetal status. CTG-net consists of three convolutional layers that extract temporal patterns and interrelationships between fetal heart rate and uterine contraction signals. We aimed to classify the abnormal group (umbilical artery pH < 7.20 or Apgar score at 1 min < 7) and the normal group from CTG data. We evaluated the performance of the CTG-net with the F1 score and compared it with conventional algorithms, namely, support vector machine and k-means clustering, and another deep neural network model, long short-term memory. CTG-net showed the area under the receiver operating characteristic curve of 0.73 ± 0.04, which was significantly higher than that of long short-term memory. CTG-net, a quantitative and automated diagnostic aid system, enables early intervention for putatively abnormal fetuses, resulting in a reduction in the number of cases of hypoxic injury.

## Introduction

Cardiotocography is a widely used examination to detect fetal status. Cardiotocograms (CTGs) comprise fetal heart rate (FHR) and uterine contraction (UC) and are continuously recorded noninvasively during labor^1^. For decades, obstetricians have diagnosed fetal status according to visual inspections of the fetal heart rate patterns in the CTG signal, such as acceleration, deceleration, baseline heart rate and heart rate variability. Additionally, the temporal relationship between FHR and UC has been widely considered to be a key factor for the interpretation of CTGs and categorized as early / variable / late / prolonged deceleration ^2^. The early deceleration is considered a result of fetal head compression and does not indicate fetal hypoxia. Variable deceleration indicates umbilical cord compression. Late and prolonged decelerations indicate deteriorated fetal status which require rapid delivery for resuscitation (Sup. Figure 1). Moreover, baseline variability also helps obstetricians to make decision for medical intervention since the reduction of baseline variability is associated with fetal hypoxic status.

To date, there are clinical problems regarding CTG: inconsistent diagnosis among obstetricians, no significant improvement in cerebral palsy rate, and a considerable false positive rate that has led to an increase in cesarean sections^3^. Even expert obstetricians interpret CTGs differently^4,5^ (interobserver variation). In addition, the same individual may give a different interpretation for the same CTG at different time points^6^ (intraobserver variation). In fact, among CTGs in cases that resulted in cerebral palsy, late deceleration suggesting uteroplacental insufficiency and variable deceleration suggesting umbilical cord compression were difficult to differentiate in 20% of cases^7^.

The objective and automated reading of CTGs has been demanded by obstetricians for a long time. Many efforts have been made to develop a CTG diagnostic aid system^8,9^, but practical performance has not been fully achieved. One of the reasons is that previous studies have mainly focused on local wave patterns (extracted features thought to represent abnormal fetal status) and have not focused on postnatal outcomes^10^. Such human-annotated abnormal features of CTG are not necessarily consistent with adverse postnatal effects. Here, we addressed the challenge of directly predicting infants’ perinatal outcomes from intrapartum CTG patterns by a DNN-based approach.

The deep neural network (DNN) is a method of machine learning composed of multiple layers that automatically extract hierarchical features, similar to the human perception mechanism^11^. Recent advancements in DNN have solved previously unachievable tasks. Today, DNN-based diagnostic systems are in a phase of clinical implementation, such as for the detection of retinal disease^12^ and skin cancer^13^. The features acquired by the DNN model are simple geometric properties (i.e., line segments) at lower levels and complex features at higher levels (e.g., FHR deceleration with temporal relation to UCs). Therefore, we hypothesized that the DNN model detects abnormal wave shapes similar to expert obstetricians. However, there is little information available for adopting DNN in CTG interpretation, especially predicting infant outcomes, compared to those in other fields.

**Figure 1:**
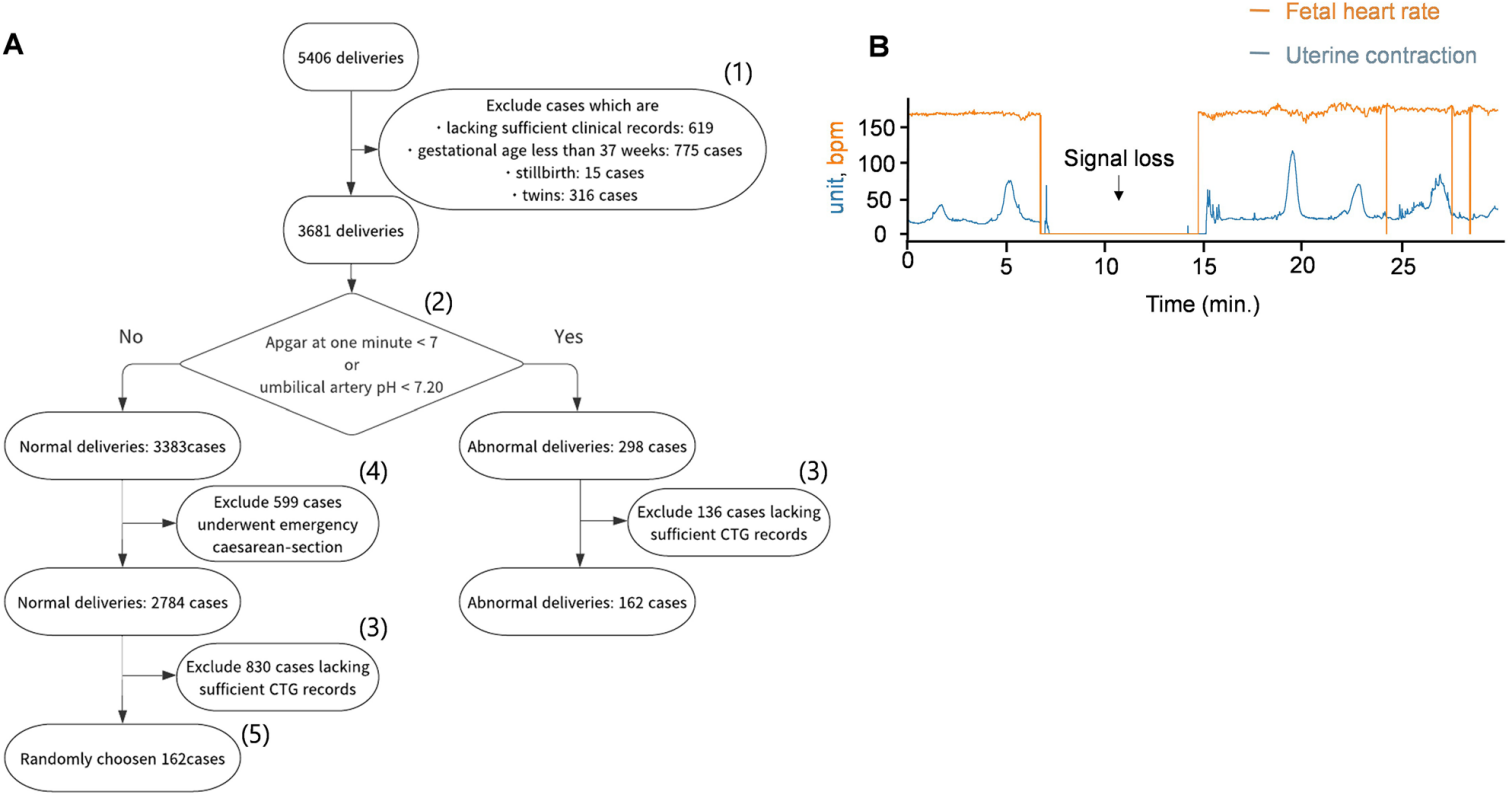
(**A**) Flowchart of data sorting. (1) Twins, stillbirths and premature deliveries were excluded. (2) The remaining 3681 data were classified into the normal and abnormal groups by Apgar score at 1 min and umbilical artery pH. (3) Deliveries lacking sufficient CTG records were excluded for both groups. (4) Normal cases that underwent emergency cesarean section were excluded. (5) A total of 162 (same as the number of abnormal cases) normal cases were randomly subsampled. (**B**) Representative figure of signal loss in FHR and UC.

In this study, we collected clinical data and the last 30 min of CTG data from 5406 deliveries took place in Keio University hospital. We cleaned the dataset with selection criteria to extract high quality samples (162 abnormal cases and 162 normal cases randomly chosen from 1954 normal cases, Fig. 1). We investigated whether DNN models could predict infant outcomes from the cleaned dataset. The architectures we adopted are convolutional neural networks (CNNs) and long short-term memory (LSTM). To validate the performance of the DNN models, we compared them with conventional machine learning algorithms, namely, support vector machine (SVM) and k-means clustering.

## Results

### Data structure and statistical features in each group

We collected clinical records for 5406 deliveries. We excluded 15 stillbirth cases, 316 twins, 775 preterm deliveries, and 619 cases lacking sufficient data. We sorted the remaining 3681 deliveries into the abnormal group if the Apgar score at 1 min was lower than 7 or the umbilical artery pH was lower than 7.20. Otherwise, deliveries were sorted into the normal group.

For the 263 abnormal deliveries, we examined the last CTGs before delivery and excluded records that were shorter than 30 min or contained more than 16% signal loss. We obtained 162 records of abnormal deliveries. We randomly subsampled an equal number of CSV records from normal deliveries that did not undergo emergency cesarean section (Fig. 1).

The umbilical artery pH was 7.31 ± 0.05 (mean ± SD, Sup. Figure 2) for the normal group and 7.25 ± 0.11 for the abnormal group. The Apgar score at 1 min was 8.31 ± 0.65 for the normal group and 4.30 ± 1.81 for the abnormal group.

### DNN outperforms the conventional algorithms

We constructed CNN-based models (CTG-net; Fig. 3, left) and LSTM-based models (Fig. 3, right). CTG-net consists of three convolution layers that extract the features in the time direction and the interaction between FHR and UC.

The performance of the models was evaluated using the F1 score (Eq. (3) in “Methods”). To confirm reproducibility, each model and algorithm were trained 10 times with different random seeds. F1 scores were 0.67 ± 0.03 (CTG-net), 0.66 ± 0.04 (LSTM), 0.55 ± 0.05 (SVM), and 0.52 ± 0.12 (k-means clustering). The performances of both DNN models were significantly higher than those of conventional algorithms (CTG-net vs. SVM: P** = 4.7 × 10^–6^, *t*_19_ = 6.4 CTG-net vs. k-means clustering: P** = 3.3 × 10^–3^, *t*_19_ = 3.4, LSTM vs. SVM: P** = 7.2 × 10^–5^, *t*_19_ = 5.1, LSTM vs. k-means clustering, P** = 6.9 × 10^–3^, *t*_19_ = 3.0, CTG-net vs. LSTM: *P* = 0.52, *t*_19_ = 0.65; *n* = 20 random seeds, Student’s *t*-test, Fig. 4A).

### CTG-net shows better performance than the LSTM model

The predictions of DNNs can be changed depending on the purpose by varying the threshold for abnormality. For example, to reduce the number of cases where abnormal situations are mistakenly overlooked, the threshold should be low. On the other hand, to reduce the cases where normal situations are mistakenly diagnosed as abnormal, resulting in unnecessary medical interventions, the threshold should be high.

To evaluate the overall performance for various thresholds, we computed a receiver operating characteristic (ROC) curve and calculated the area under the curve (AUC). The AUC of CTG-net (raw data, Fig. 4B) was 0.73 ± 0.04, and the AUC of the LSTM-based model (raw data, Fig. 4B) was 0.62 ± 0.03. CTG-net showed a significantly higher AUC than the LSTM-based model (P** = 4.8 × 10^–5^, *t*_19_ = 5.3, *n* = 20 random seeds, Student’s *t* test).

To improve the prediction accuracy, each CTG-net and LSTM-based model was trained with preprocessed smoothed data. However, the model trained with raw data tended to show slightly higher performance than models trained with smoothed data in both DNN models (CTG-net: *P* = 0.20, *t*_19_ = 1.3, LSTM-based model: *P* = 0.84, *t*_19_ = 0.19, *n* = 20 random seeds, Student’s *t* test). The AUC of CTG-net (smoothed data, Fig. 4B) was 0.70 ± 0.03, and the AUC of the LSTM-base model (smoothed data, Fig. 4B) was 0.62 ± 0.02.

### DNNs showed consistent performance utilizing novel data acquired after training

Finally, we tested the performance of the models with the data of 380 deliveries (363 normal and 17 abnormal deliveries after excluding cases as in the protocol shown in Fig. 1) collected from Apr. 2019 to Oct. 2020, which were never used in training or validation of the models. The AUC of CTG-net was 0.71 ± 0.04 and LSTM model was 0.61 ± 0.02. CTG-net showed a significantly higher AUC than the LSTM-based model (P** = 1.3 × 10^–6^, *t*_19_ = 7.1, *n* = 20 random seeds, Student’s *t*-test). Representative confusion matrix and AUC-ROC are shown in Sup. Figure 4.

### CTG-net showed the same level of prediction accuracy for different datasets (CTU-UHB)

CTU-UHB Intrapartum Cardiotocography Database is a widely used dataset to study CTGs^14^ dataset. CTU-UHB data is consists of 552 births in 2 years. CTG-net was applied to classify CTU-UHB data. According to our definition, the normal deliveries were 354 cases and abnormal ones were 198 cases. 52 normal and 26 abnormal cases which satisfied the selection criteria were used for analysis. CTG-net trained with Keio University Hospital data showed ROC-AUC 0.68 ± 0.03. There is no significant difference between the ROC-AUC for Keio data (Sup. Figure 5, *P* = 0.06, *t*_19_ = 2.0, *n* = 20 random seeds, Student’s *t*-test).

## Discussion

In this study, we demonstrated that DNN models could predict infant outcomes from the last 30 min of CTG just before delivery. Both F1 score and ROC-AUC of the newly developed DNN models were significantly better than those of the other algorithms we tested. In addition, we clarified that the CTG-net model achieved significantly higher performance than the LSTM model. Although the model predictions are not completely accurate, automatic prediction of postnatal health condition in advance without bias can be helpful to obstetricians in determining the need for medical interventions.

Obstetricians visually classify CTGs into several categories based on significant wave patterns according to the guidelines^1^. In clinical practice, obstetricians have been able to recognize specific patterns (acceleration, deceleration, baseline heart rate, heart rate variability) in CTGs and use them to make decisions about necessary medical interventions, such as cesarean section. Some authors argue that there are limitations to making accurate diagnosis with such human visual-based CTG^3,6^. Therefore, a quantitative and objective algorithm for CTG evaluation has been demanded for decades. In the present study, we found that it is possible to predict the presence or absence of acidemia in infants by analyzing CTG patterns using DNNs without any preprocessing. These results suggest that some elements of the CTG, traditionally dismissed, could have valuable contents that indicate perinatal outcomes.

In our study, the DNN models outperformed the conventional algorithms in F1 score (Fig. 4A). Since SVM and k-means are not suitable for high-dimensional input, dimensionally reduced features, such as the number of early accelerations and severity of deceleration, are fed to the algorithms. However, it is possible that these human-biased features miss critical information for classifying normal and abnormal features. DNNs can be trained with high-dimensional raw traces without overfitting due to dropout layers^15^. Therefore, DNNs outperformed the conventional algorithms. The DNNs tended to show higher performance when trained with raw data than with the denoised and smoothed data (Fig. 4B). This indicates that the reduced information previously considered noise might contribute to performance improvement. Since DNNs themselves have average pooling layers that function as denoising and smoothing, the advantage of CTG-net is that it does not require preprocessing.

CNN-based CTG-net outperforms LSTM in ROC-AUC (Fig. 4B) despite the number of parameters in the CTG-net (2130) being smaller than that of the LSTM (483,602). This indicates that the complexity of the CTG-net is lower than that of the LSTM. A possible reason is that the CNN models utilize broader temporal information for prediction. The CNN models acquire various filters of 30-s bins (kernel size = 30) to extract temporal abnormal shapes of CTGs, whereas LSTM models update their internal states every 1 s. We assume that the 30-s bin is long enough to extract features such as baseline variability, deceleration, and acceleration. At the second level, the depthwise convolution layer represents the relationship of the contemporary FHR and UC. These features are integrated at a higher level. Therefore, it is possible to differentiate early, variable, late, and prolonged deceleration, as well as to extract complex features such as waveforms that show deceleration but no variability (suggesting more severe acidemia)^16^. This stepwise reading of waveform patterns is similar to the obstetrician's reading procedure and is equivalent to subclass classification in guidelines. To extract these features, we used 4–8 filters in the convolution layers. It is assumed that this model design is the reasons why CTG-net has shown superiority over LSTM.

DNNs do not always perform equally well in different recording environments and with data from patients of different backgrounds. Therefore, we tested CTG-net and LSTM model with novel data (Apr. 2019–Oct. 2020) obtained after model training. Because of the imbalanced dataset, we showed confusion matrix. Both models show higher recall (number of abnormal cases correctly classified as abnormal) than 0.7. The goal of this study is to reduce the number of FN, this result is consistent with our objective. Furthermore, the AUCs were equivalent to those of the previous data (Apr. 2011–Mar. 2019) in our hospital and open access CTU-UHB dataset^14,17^ (Sup. Figure 5).

In a previous study, a CNN-based model was trained with a larger dataset and showed an AUC of 0.68^18^, which was slightly lower than ours (AUC 0.73). The abnormal group was defined as cases with umbilical artery pH less than 7.05, which is a lower threshold than ours (less than 7.20). Fetal acidemia is correlated with deceleration area^19,20^. If the deceleration is deeper and wider, infants tend to show more severe acidemia. Thus, the classification task with our dataset is relatively more detailed than the previous study setting.

We tried to reduce the number of parameters to avoid overfitting. We adopted a model with only three layers. We also utilized a separable and depthwise convolution layer^21^, which has a small number of parameters but does not degrade the performance. Many of the models used in recent machine learning studies consist of tens or hundreds of layers^22,23^, which leads to a higher risk of overfitting, whereas our model only has 3 simple and sophisticated layers. Moreover, our model has a depthwise separable convolution layer, which reduces the number of parameters without decreasing the representational power^21^.

The amount of data samples is very small for a DNN training in general. However, we achieved better classification accuracy with CTG-net and LSTM as compared to SVM and k-means clustering. There are four possibilities as reasons; (1) The models were trained with highly qualified samples which satisfied several selection criteria (Fig. 1). (2) Our task is binary classification (normal and abnormal) that requires less samples than multiclass classification. (3) The size of the model is smaller than general DNN architectures (Fig. 3). (4) We adopted dropout to avoid overfitting.

In the clinical setting, an AUC value from 0.7 to 0.9 is considered to indicate moderate accuracy^24^. We believe that our model (AUC 0.73) has the potential to achieve clinically acceptable accuracy and reliability. However, among the various models we applied, the maximum AUC was 0.77, and it seems difficult to predict with nearly 100% accuracy. These results suggest that there is a limitation on the amount of information derived from the original CTG data. CTG only consists of the two signals (FHR and UC) with 4 data points in 1 s (4 Hz). Multiple electrodes and a high sampling rate could make it possible to implement automatic diagnostic aid systems in clinical settings, such as electrocardiograms (12-lead, 1000 Hz)^25^ and electroencephalograms (19-channel, 2000 Hz)^26,27^. Their spatiotemporal resolutions are much larger than those of CTGs. To overcome these difficulties in the classification task using CTGs, research in this field may be required to develop equipment to obtain good-quality CTG data with multiple electrodes and a high sampling rate, similar to other fields.

In conclusion, in this study, we demonstrated that our CTG-net could predict infant outcomes from the last 30 min of CTG just before delivery with clinically acceptable accuracy and reliability. By constructing the model with high performance, we are able to apply it to practical use. The automated diagnostic aid system for CTGs can be used as (1) an early alert to reduce the development of fetal acidemia and cerebral palsy by detecting abnormal patterns that humans cannot recognize, (2) an evaluation system for the retrospective discussion of normal/abnormal cases as a quantitative metric without human biases, and (3) an automated reassurance system for putative normal fetal status, which may reduce the burden of routine work for obstetricians and caregivers/midwives.

## Methods

### Data acquisition

CTGs of 5406 deliveries were recorded in the Keio University Hospital from Apr. 2011 to Oct. 2020. This study was conducted according to the guidelines of the Declaration of Helsinki, and all procedures were approved by the ethics committee of the School of Medicine, Keio University, in Aug. 2019 (Accession No. 2019-0089). Due to the retrospective design of this study, opt-out informed consent was employed. All information about the patient is processed anonymously in accordance with the De-Identification Standard of the HIPAA Privacy Rule.

### Data sorting

We obtained a total of 3681 clinical records after excluding 15 stillbirths, 316 twins, 775 premature births, and 619 cases that lacked sufficient records (Fig. 1). We classified cases that showed umbilical artery pH lower than 7.20 or Apgar score lower than 7 at 1 min as abnormal; otherwise, cases were classified as normal.

We obtained a total of 298 abnormal cases, of which 136 cases were excluded (45%) because of a short duration of the CTG record (less than 30 min) or excessive signal loss (more than 16% of the signal). As a result, 162 cases were used for model training and validation.

For the normal group, we excluded 617 patients who underwent emergency cesarean sections. Out of the 2873 normal cases, we randomly chose 162 cases to organize the training dataset to have an equal number of normal and abnormal cases.

### Inspecting representative CTG images by eye

**Figure 2:**
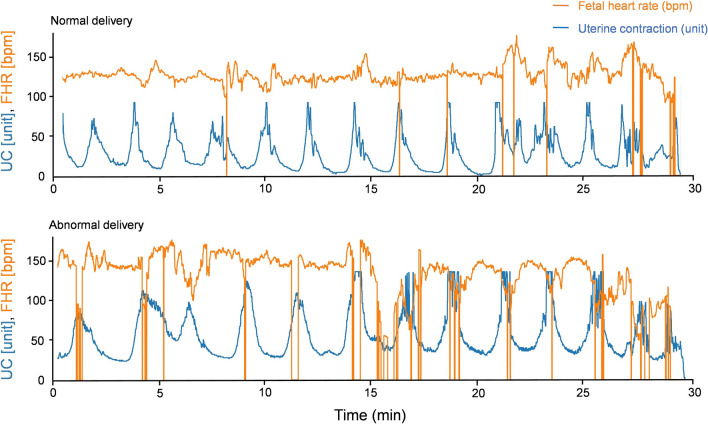
Representative raw CTG records from the normal and abnormal deliveries. Top: CTG records from a normal delivery. Bottom: CTG records from an abnormal delivery. The orange line represents fetal heart rate (bpm), and the blue line represents uterine contraction (unit).

Figure 2 displays representative CTG images of the normal and abnormal groups. In the normal waveform (*top*), acceleration is frequently observed, and the rise from the baseline is apparent. Transient variable deceleration is observed just before delivery, suggesting that fetal status is reassured. In the abnormal waveform (*under*), a decrease in FHR synchronized with a rise in UC (early deceleration; not an indicator of non-reassuring fetal status) was observed in the first half of the waveform. A delayed decrease in FHR to the peak of UC (late deceleration) and a deep and wide decrease in FHR (prolonged deceleration) were observed in the latter half. Both signs are indicators of non-reassuring fetal status, suggesting the development of fetal hypoxia.

### Preprocessing for the conventional algorithms

Using custom-made MATLAB code (MathWorks, Natick, MA, USA), we constructed nonlinear SVM^28^ and k-means clustering^29^ algorithms to classify normal and abnormal groups using the human extracted features. Conventional algorithms cannot be applied to raw data since the dimensionality of the data is too high. The data need to be preprocessed and transformed into low-dimensional features.

Preprocessing consists of denoising, smoothing, Hilbert transform and peak detection steps^8^. Sup. Figure 3 shows the processing procedure. First, visually evident noises that are physiologically implausible were replaced by the average of the values before and after them (Sup. Figure 3, 1st and 2nd row). Next, waves were smoothed by a bilateral moving average of 15 points (Sup. Figure 3, 3^rd^ row). A Hilbert transform was performed for the smoothed waves^30^, and the onset/offset of the acceleration and deceleration were identified (circles in Sup. Figure 3, 3rd row). Then, the number of accelerations and the total area of the decelerations in each case were calculated. Algorithms were trained with these features.

### Deep neural network implementation

We proposed two types of DNN models: CNN-based models and LSTM-based models. Both models are widely used for time series data. Models were trained on an Intel Core i7-8650U CPU at 1.90 GHz in TensorFlow^31^ using the Keras API^32^.

**Figure 3:**
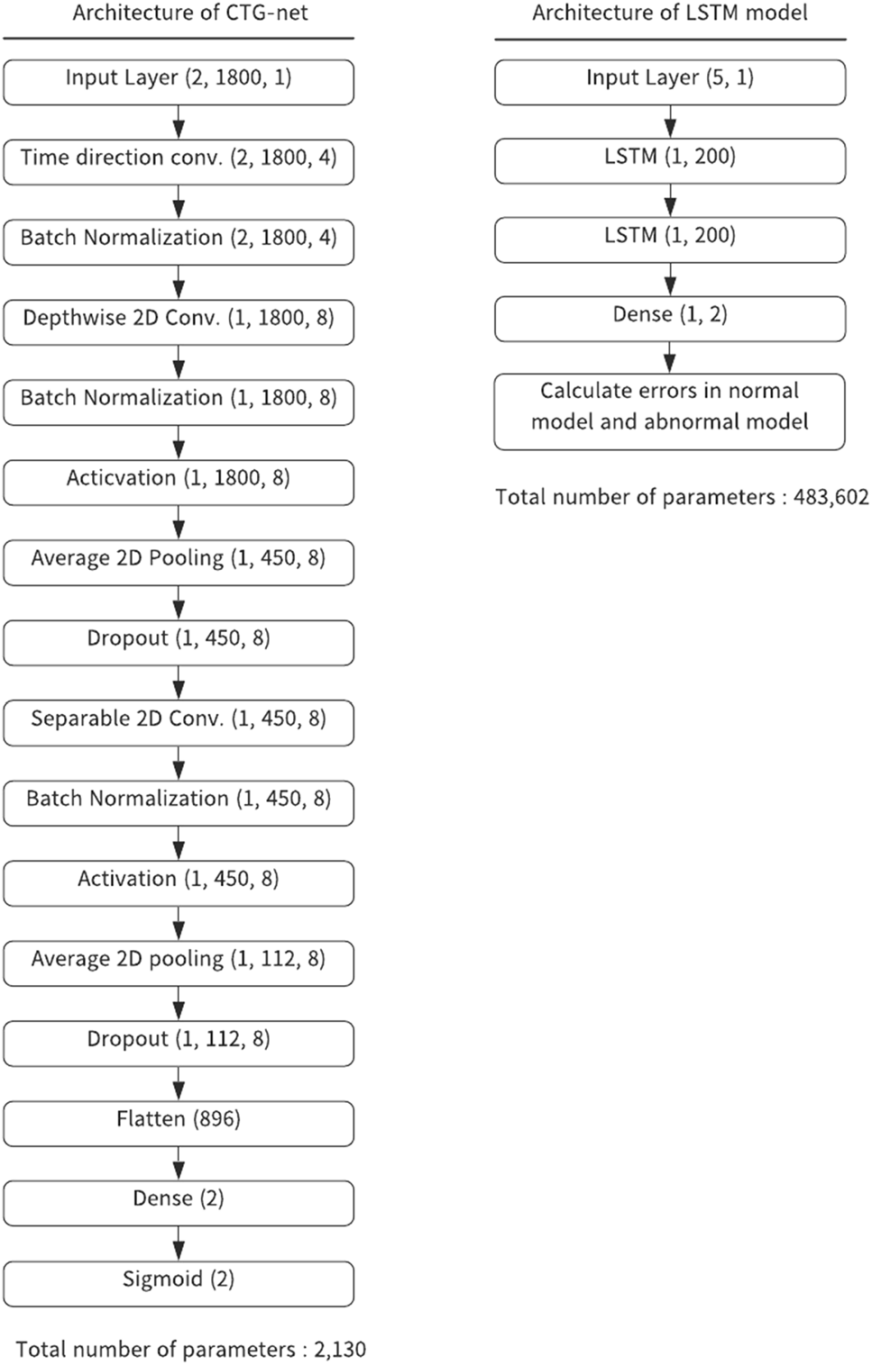
Architecture of DNN models. Left: The CTG-net is a CNN-based model with 3 convolutional layers. The model outputs abnormality and normality with values of 0–1. Right: The LSTM consists of 2 layers with 40 recurrent units. Input to the model is 5 consecutive data points of FHR and UC signals. The model predicts the next value of FHR and UC.

The architecture of the DNN models is shown in Fig. 3. The left part represents a CNN-based model, which we named CTG-net. The CTG-net takes FHR and UC signals of 1800 time points (downsampled at 1 Hz for 30 min) as input. The input data were convolved with 30-s-bin kernels in the time direction. This 30-s-bin corresponds to the duration of the visual inspection to detect non-reassuring fetal status by obstetricians (Sup. Figure 1). For instance, the 30-s FHR is used as a criterion to distinguish the variable deceleration and the late deceleration.

Then, batch normalization^33^ was conducted. The second convolution among signals is performed to learn the relationship between FHR and UC (i.e., acceleration can be detected when the values of both signals increase simultaneously). The second batch normalization followed by activation, average 2D pooling, and dropout^15^ was conducted. The activation layer is the nonlinear function that transforms a sum of inputs from the previous layer. We applied an exponential linear unit (ELU) as an activation function in the CTG-net since it achieved better performance than a rectified linear unit (ReLU)^34^. The pooling layer summarizes the neighboring values in the same kernel into the average value, which contributes to the reduction of the computational cost and makes the model robust to the temporal shift^35^. The dropout layer randomly inactivates a certain percent of the units in the layer to avoid overfitting. We set the dropout rate to 25% in the CTG-net. The third convolution, separable convolution, was performed on the time series signals, reflecting the relationship between FHR and UC obtained by these operations. This layer convolves in the time and kernel directions independently, which enables integrated feature extraction with fewer parameters. This results in the model recognizing the more detailed change in long-term trends, such as early deceleration and late deceleration, by comparing the difference of peaks in FHR and UC. The third batch normalization and the second activation, pooling, and dropout layers were added before the flatten layer, which aligns 3-dimensional inputs into 1-dimensional outputs to utilize all features for classification. The last layer is a dense layer with sigmoid activation that outputs abnormality (normality is calculated as 1 − abnormality). The total number of parameters was 2130. Previous studies investigated several optimizers for analyzing biological signals, and Adam was considered the best^36^. We used Adam optimizer with a learning rate of 10^–3^ and epsilon of 10^–5^^37^. We adopted categorical cross-entropy as a loss function. The *abnormality* was used to classify normal and abnormal cases, then the model was evaluated with F1 score. By varying the threshold of *abnormality*, we investigate the trade-off between the recall and the precision in each threshold, resulting in ROC-AUC.

We implemented the long short-term memory model using the same libraries as CTG-net (TensorFlow using the Keras API). We split the FHR and UC signals into 5 consecutive time points as an input and the next value as a true output. The task for the model is to predict the true output from the input signals. The LSTM model consists of 2 layers with 40 recurrent units (Fig. 3, right). We applied the rectified linear unit as an activation function, followed by the dense layer, which outputs the predicted FHR and UC values. The total number of parameters was 483,602.

We trained an LSTM model with 162 abnormal CTG data and trained another LSTM model with an equal number of normal CTG data. When test CTG data are fed to the models, each model gives a predicted value. After subtracting the true value from each value, we can compare the absolute error from each model. We summed the errors with the 5-s bin window in each model. When the total error was smaller in the normal model, we assumed that the test CTG data were from a normal delivery, and vice versa. We calculated the difference in errors from each model (*Risk index*_*LSTM*_ = *Error*_*normal *_*– Error*_*abnormal*_), and by varying the threshold, the test data were classified as normal or abnormal. Therefore, we computed the ROC curve and calculated the AUC.

**Figure 4:**
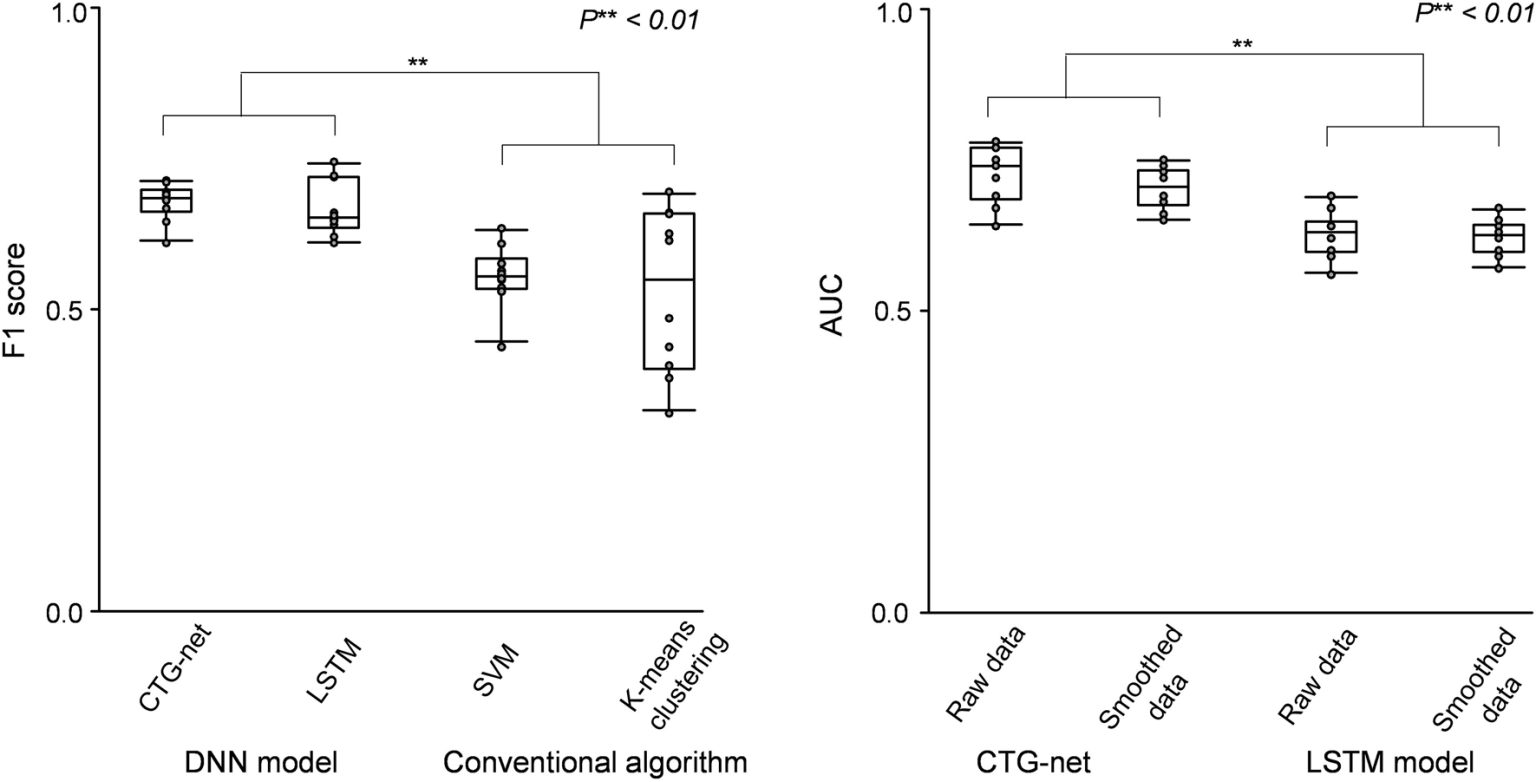
(**A**) F1 score of CTG-net, LSTM-based model, SVM, and k-means clustering. (**B**) ROC-AUC of the CTG-net and LSTM-based models trained with raw data or smoothed data.

We tested CTG-net and LSTM-based models with tenfold cross validation for raw CTG data and smoothed CTG data (Fig. 4B). The amount of normal and abnormal data was fixed throughout the experiment. The 162 normal data were sampled 10 times without replacement from 1954 deliveries which satisfied the selection criteria (Fig. 1(5)). All 162 abnormal data were resampled 10 times so that each of the split sample groups was a test case once. The normal and abnormal data were divided into training and test sets at 9:1 respectively.

### Evaluation of the models

The performance of the conventional algorithms and DNN models was compared by F1 score, a harmonic average of precision and recall. The F1 score was calculated from precision and recall as follows:

1 $$Precision = \frac{{TP}}{{TP + FP}},$$

2 $$Recall = \frac{{TP}}{{TP + FN}}$$

3 $$F1\;score = \frac{{2 \times precision \times recall}}{{precision + recall}}$$

where TP, FP, and FN represent true positives, false positives, and false negatives, respectively. Positive corresponds to abnormal and negative corresponds to normal.

The performance of the CTG-net and LSTM-based models were compared by ROC-AUC, which is computed with various thresholds. When the threshold is set low (sensitive to abnormalities), TP and FP increase, resulting in better recall and worse precision, and vice versa when the threshold is set high. We calculated the AUC for CTG-net with *abnormality *(*CNN*) and for LSTM with *Risk index*_*LSTM*_. P* represents p-value < 0.05; P** represents p-value < 0.01.

### Evaluation of model generalization to another dataset

552 samples from CTU-UHB Intrapartum Cardiotocography Database were divided into 354 normal cases and 198 abnormal cases according to our definition. Repeated zero signals at the last of samples were removed, and the 30 min before the last nonzero signals were extracted. 52 normal and 26 abnormal cases which satisfied the selection criteria (signal loss less than 16%) were used for analysis.

## Supplementary Information

Supplementary Figure 1.

Supplementary Figure 2.

Supplementary Figure 3.

Supplementary Figure 4.

Supplementary Figure 5.

Supplementary Information.

## Data Availability

The data that support the findings of this study are available from the corresponding authors upon reasonable request. Source data are provided with this paper.
