# Supplementary figures and images for: Deep neural network-based classification of cardiotocograms outperformed conventional algorithms
Source: Sci Rep. 2021 Jun 28;11:13367. doi: 10.1038/s41598-021-92805-9 (PMC8238938; doi:10.1038/s41598-021-92805-9)

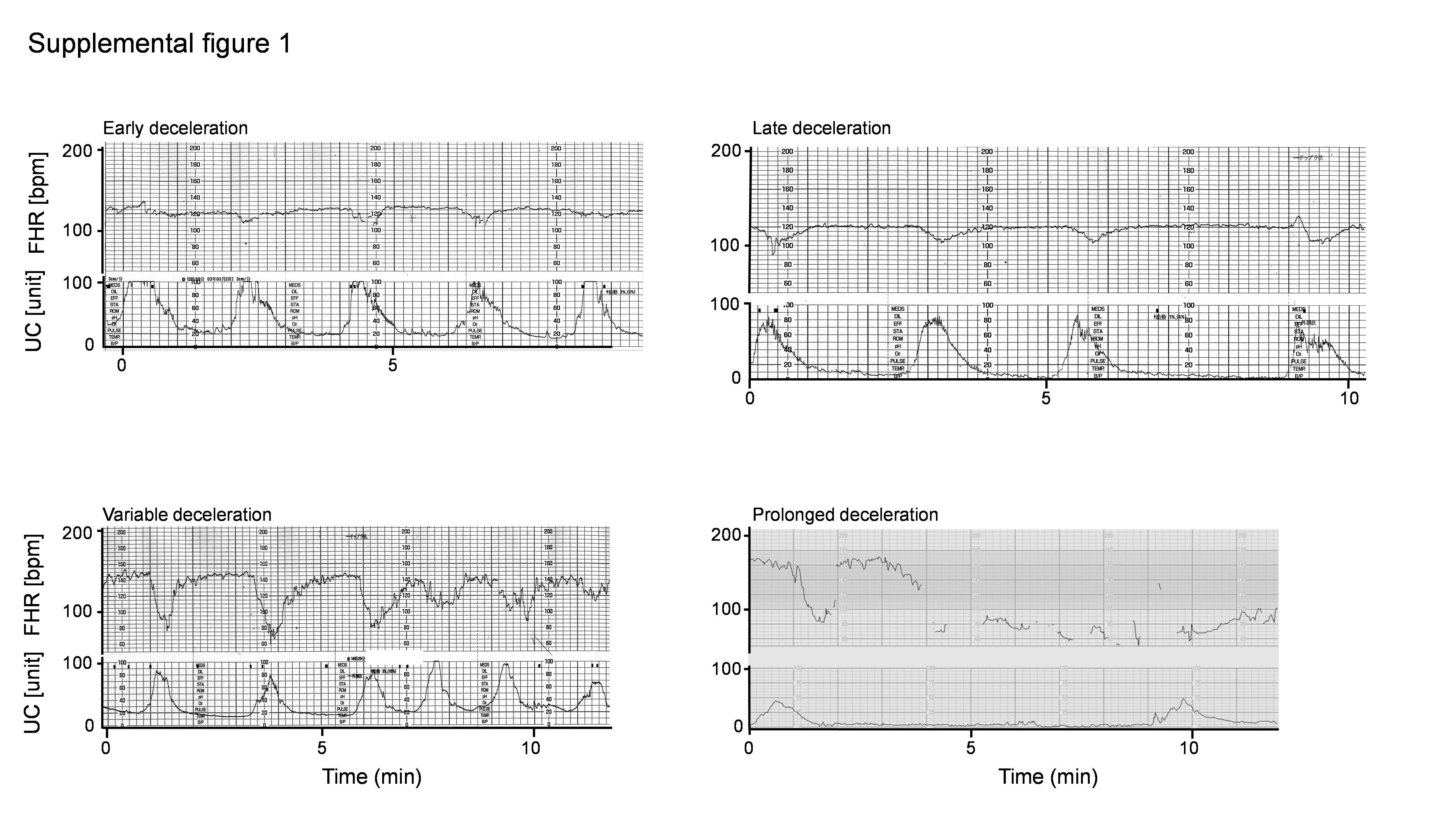

Supplement: Supplementary file 1 — Supplementary Figure 1. [file 41598_2021_92805_MOESM1_ESM.jpg]

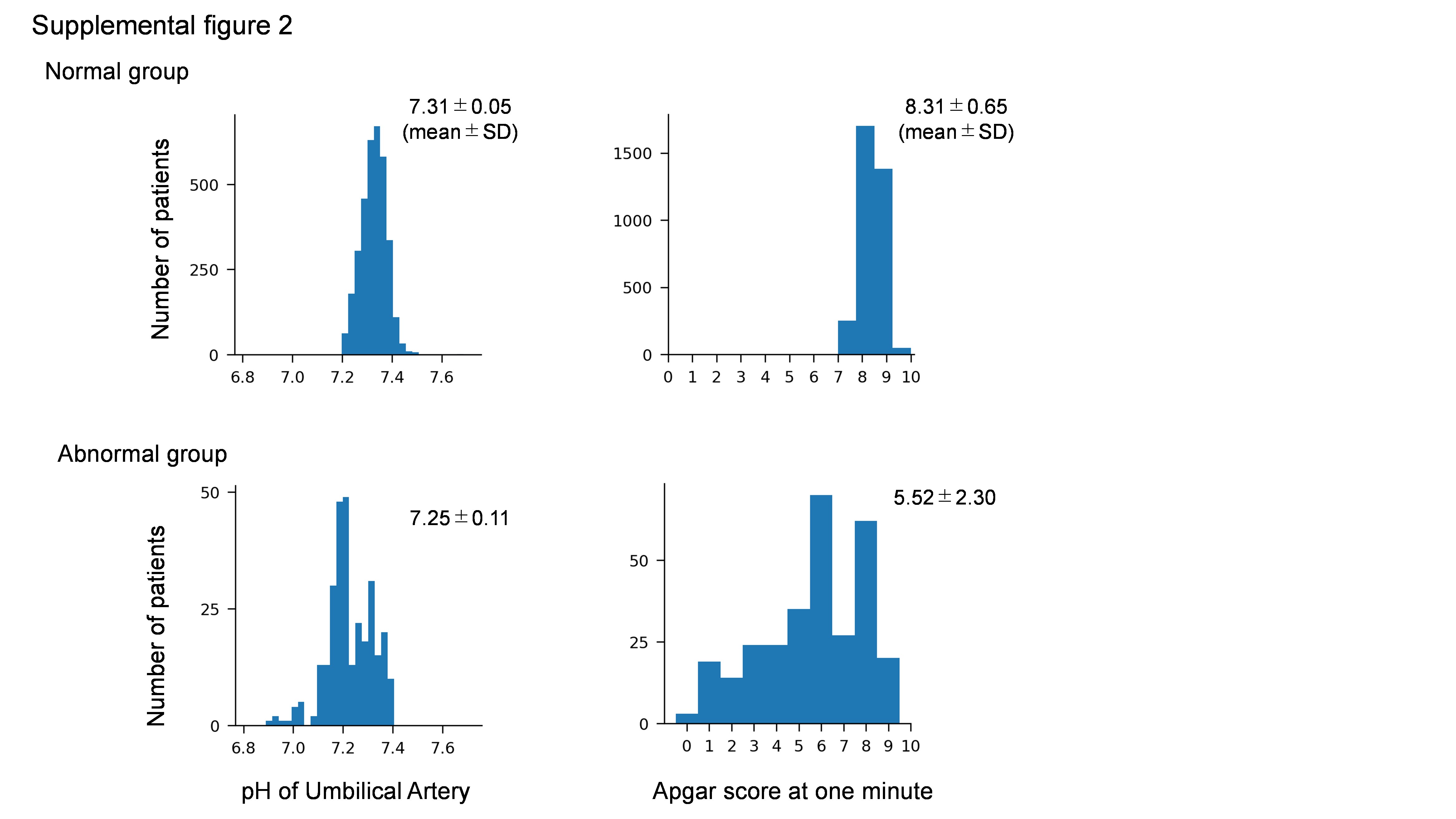

Supplement: Supplementary file 2 — Supplementary Figure 2. [file 41598_2021_92805_MOESM2_ESM.jpg]

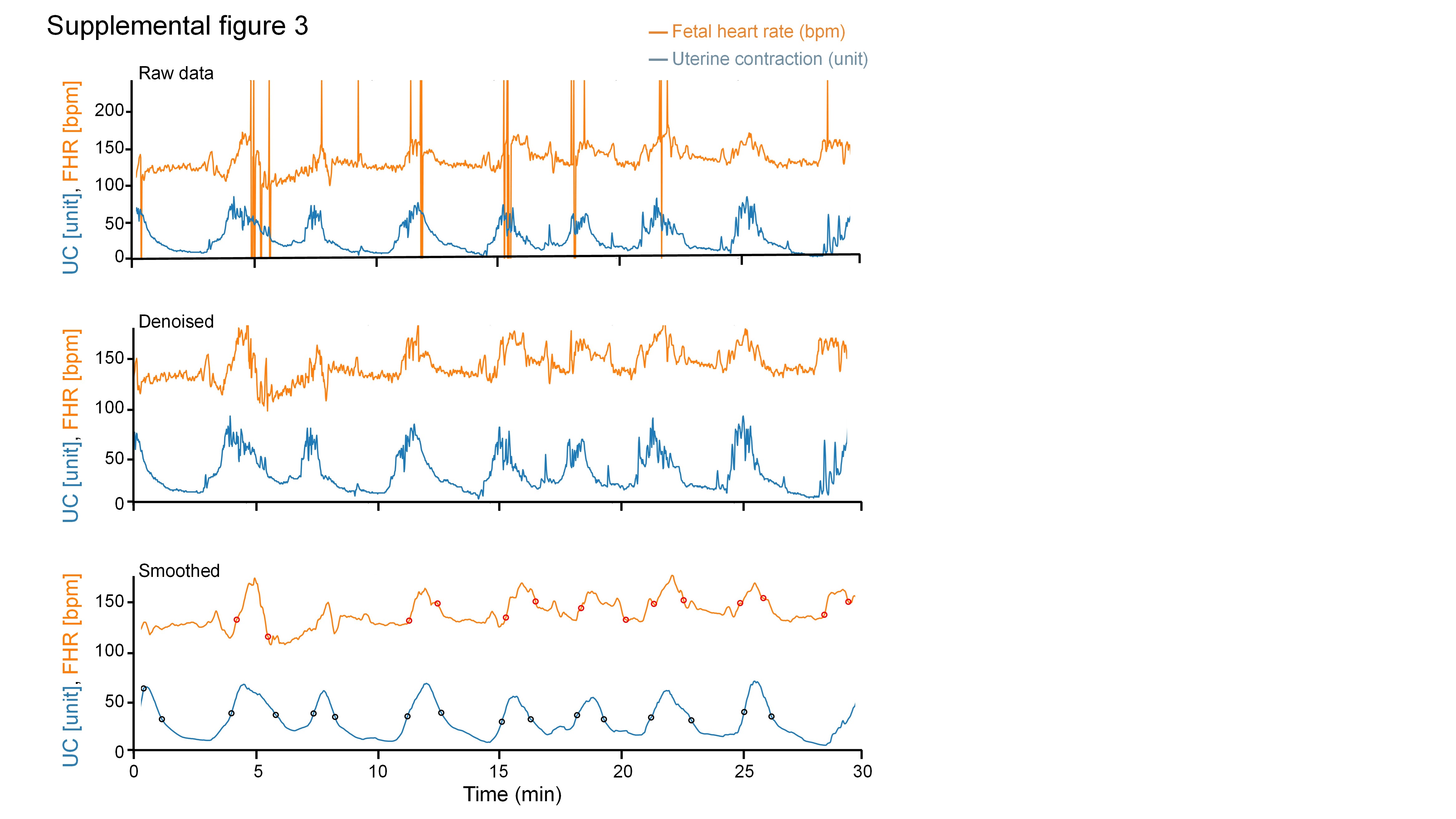

Supplement: Supplementary file 3 — Supplementary Figure 3. [file 41598_2021_92805_MOESM3_ESM.jpg]

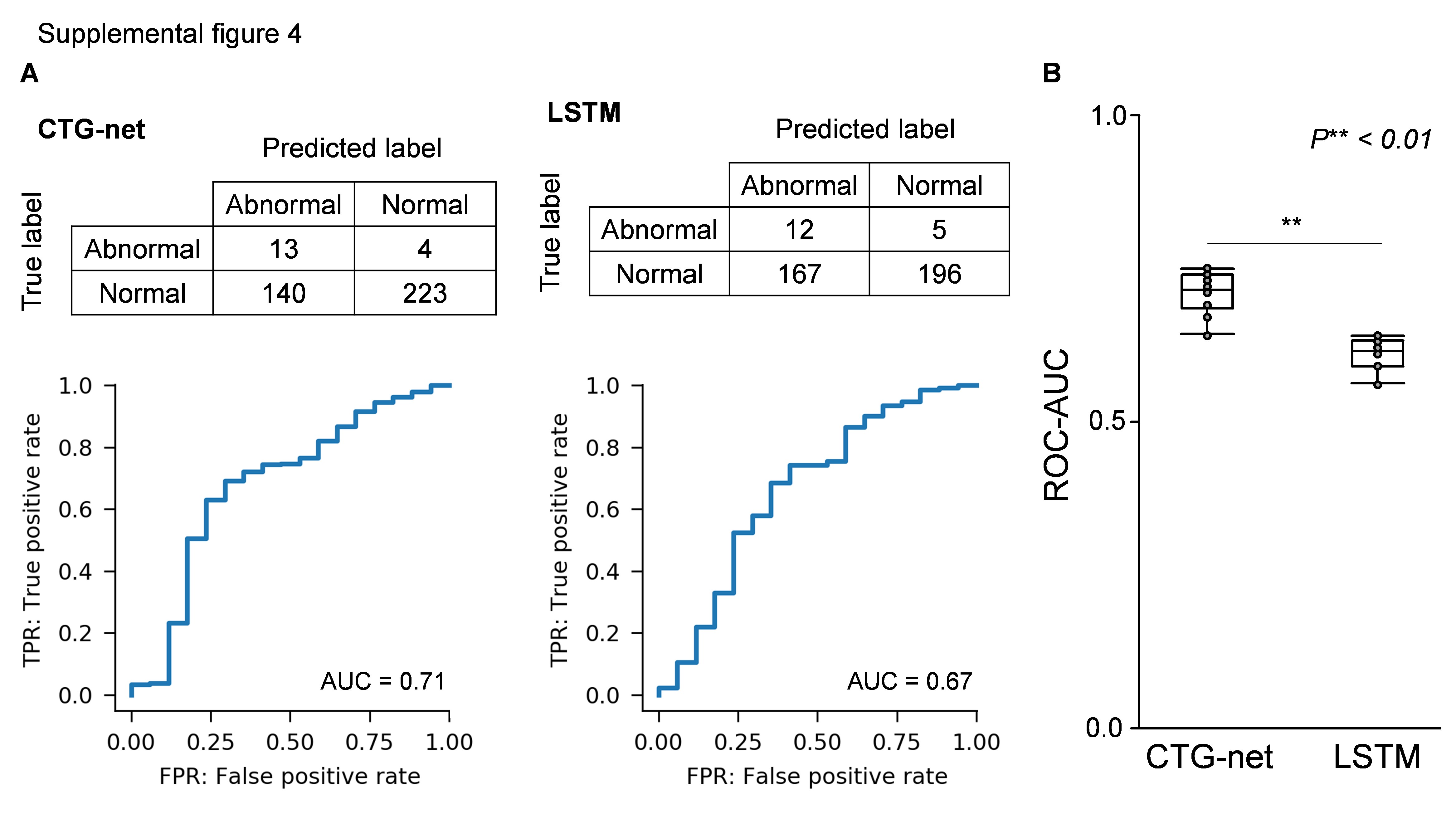

Supplement: Supplementary file 4 — Supplementary Figure 4. [file 41598_2021_92805_MOESM4_ESM.jpg]

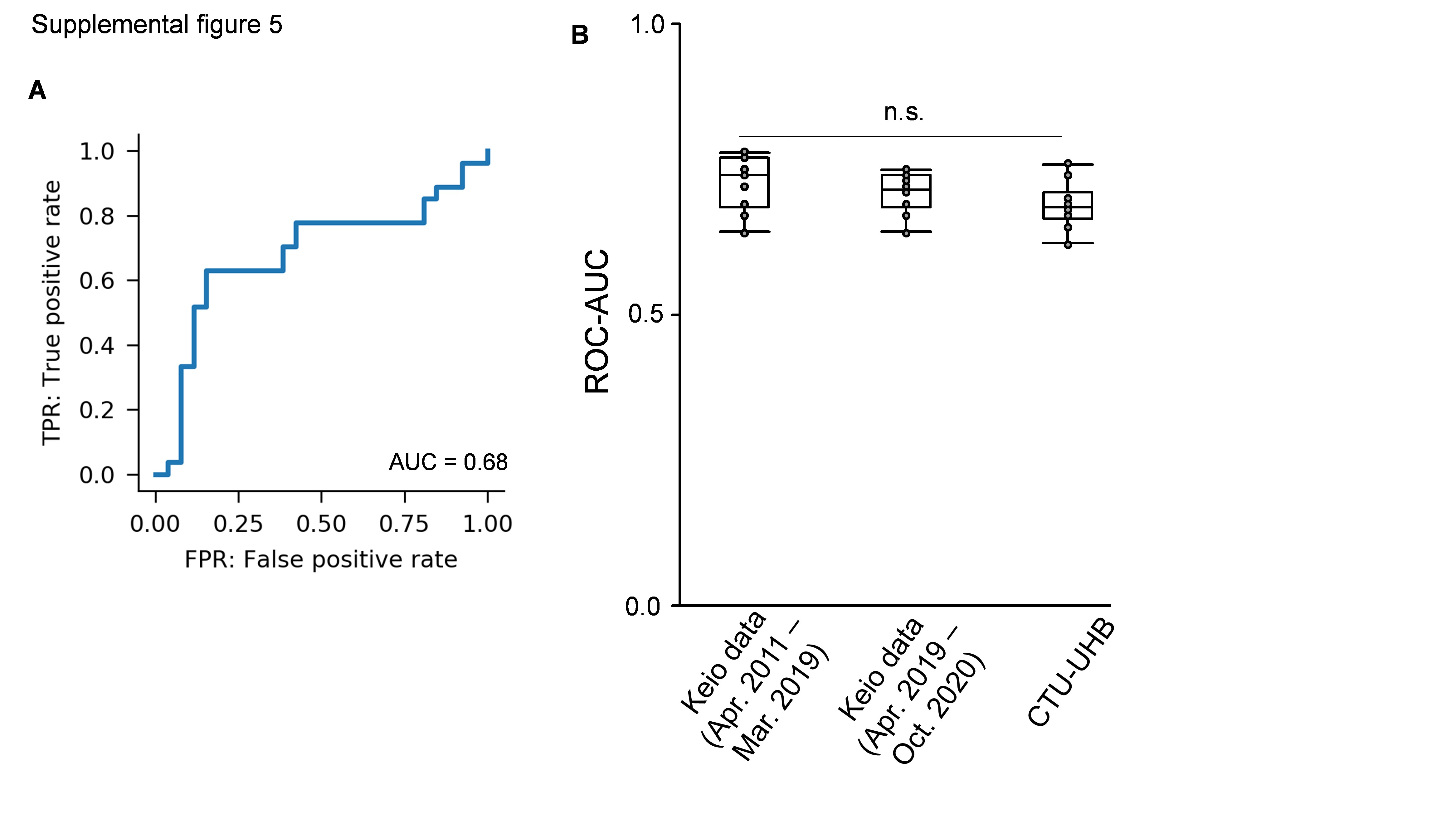

Supplement: Supplementary file 5 — Supplementary Figure 5. [file 41598_2021_92805_MOESM5_ESM.jpg]
