# Supplementary material for: Deep neural network-based classification of cardiotocograms outperformed conventional algorithms
Source: Sci Rep. 2021 Jun 28;11:13367. doi: 10.1038/s41598-021-92805-9 (PMC8238938; doi:10.1038/s41598-021-92805-9)
Supplement: Supplementary file 6 — Supplementary Information. [file 41598_2021_92805_MOESM6_ESM.docx]

Supplemental figure 1: The original CTG trace on paper roll. *Top left*) early deceleration exhibits shallow and short-lasting deceleration that is coincident with UC. It is considered a result of fetal head compression and does not indicate fetal hypoxia. *Bottom left*) Variable deceleration is a rapid decrease (reaching the nadir within 30 seconds) and recovery with various amplitude, shape and relationship to UC due to umbilical cord compression. *Top right*) Late deceleration is a U-shaped gradual decrease, and recovery (more than 30 seconds to/from the nadir) starts more than 20 seconds after the onset of UC; it is a sign of chemoreceptor-mediated response to fetal hypoxia. *Bottom right*) Prolonged deceleration consists of an apparent decrease in FHR from the baseline over 15 bpm and persisting more than 2 minutes in duration, which indicates acute fetal hypoxia

Supplemental figure 2: Histogram of the umbilical artery pH (*left*) and Apgar score at one minute (right) of the normal group (*top*) and abnormal group (*bottom*).

Supplemental figure 3: The preprocessing procedure consists of denoising, smoothing, Hilbert transform and peak detection steps. *Top*: Raw FHR (*orange*) and UC (*blue*) waves. *Middle*: Noises were replaced by the average of the values before and after them. *Bottom*: Signals were smoothed by a bilateral moving average of 15 points. The onset/offset of the acceleration and deceleration (*circles*) were identified by Hilbert transform.

Supplemental figure 4: A) Confusion matrix (*top*) and ROC-AUC (*bottom*) of the CTG-net (*left*) and LSTM model (*right*) for the novel dataset (normal: 363, abnormal: 17 samples). B ROC-AUC of CTG-net and LSTM-based models for the novel data.

Supplemental figure 5: A) Representative ROC-AUC of CTG-net trained with the Keio university hospital dataset for CTU-CHB dataset. B) ROC-AUC of CTG-net (trained with Keio University Hospital dataset) for Keio test data (Apr. 2011 - Mar. 2019, Apr. 2019 - Oct. 2020) and CTU-UHB dataset.
